# *SDMtune*: An R package to tune and evaluate species distribution models

**DOI:** 10.1002/ece3.6786

**Published:** 2020-09-30

**Authors:** Sergio Vignali, Arnaud G. Barras, Raphaël Arlettaz, Veronika Braunisch

**Affiliations:** ^1^ Division of Conservation Biology Institute of Ecology and Evolution University of Bern Bern Switzerland; ^2^ Forest Research Institute of Baden‐Wuerttemberg Freiburg Germany

**Keywords:** ecological niche model, fine‐tuning, genetic algorithm, machine learning, model complexity, variable selection

## Abstract

Balancing model complexity is a key challenge of modern computational ecology, particularly so since the spread of machine learning algorithms. Species distribution models are often implemented using a wide variety of machine learning algorithms that can be fine‐tuned to achieve the best model prediction while avoiding overfitting. We have released *SDMtune*, a new R package that aims to facilitate training, tuning, and evaluation of species distribution models in a unified framework. The main innovations of this package are its functions to perform data‐driven variable selection, and a novel genetic algorithm to tune model hyperparameters. Real‐time and interactive charts are displayed during the execution of several functions to help users understand the effect of removing a variable or varying model hyperparameters on model performance. *SDMtune* supports three different metrics to evaluate model performance: the area under the receiver operating characteristic curve, the true skill statistic, and Akaike's information criterion corrected for small sample sizes. It implements four statistical methods: artificial neural networks, boosted regression trees, maximum entropy modeling, and random forest. Moreover, it includes functions to display the outputs and create a final report. *SDMtune* therefore represents a new, unified and user‐friendly framework for the still‐growing field of species distribution modeling.

## Graphical abstract

The main innovations of *SDMtune* are a novel genetic algorithm to tune the hyperparameters of a model and functions to perform data‐driven variable selection. Real‐time and interactive charts are displayed during the execution of several functions to help users understand the effect of removing a variable or varying model hyperparameters on model performance.

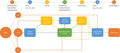

## 1 INTRODUCTION

Species distribution models (SDMs) encompass a variety of methods used to predict the occurrence of a species from the environmental conditions at a given site, thus providing a proxy of habitat suitability (Warren & Seifert, 2011). These methods are increasingly used in various fields of ecology (Guisan & Thuiller, 2005), often with the aim of guiding decision‐making in species conservation management and planning (Guisan et al., 2013). Indeed, SDMs represent a crucial and cost‐effective tool to identify current important areas for threatened species, and to forecast ecosystem impacts of rapid human‐induced environmental change (Beaumont et al., 2016; Elith, Kearney, & Phillips, 2010; Franklin, 2013; Guillera‐Arroita et al., 2015; Guisan et al., 2013; Schwartz, Iverson, Prasad, Matthews, & O’Connor, 2006). Among the variety of available algorithms, machine learning approaches are becoming increasingly popular, facilitated by the recent availability of high computational power, and due to their ability to fit complex nonlinear relationships without requiring an a priori definition of a data model (Breiman, 2001). However, there still are many decisions to be made at various steps of the model building process that can influence the final output (Guisan & Thuiller, 2005). For example, the amount of complexity should be cautiously controlled to avoid models that underfit or overfit the underlying data (Merow et al., 2014; Warren & Seifert, 2011).

In general, the amount of complexity of a model depends on the number of chosen predictors and their transformations (Merow et al., 2014). Moreover, each machine learning algorithm has a series of parameters, known as hyperparameters. In contrast to model parameters, which are estimated from the data during model training, hyperparameters have a fixed value that must be defined before model training. Even if most machine learning algorithms have predefined default values, the optimal value of each hyperparameter is unknown, as it is specific to the modeling problem and the dataset. However, its choice affects model complexity and/or performance. For example, in a neural network, the maximum number of iterations controls the amount of iterations executed by its optimization algorithm. This value does not affect model complexity but if it is too low the algorithm might not converge, thus generating a model with lower performance. On the other hand, increasing the size of the hidden layer increases the number of parameters of the model and consequently its complexity, which in turn might affect its performance. In a Maxent model (Phillips, Anderson, & Schapire, 2006), the amount of regularization controls overfitting by shrinking some parameters toward zero which consequently penalizes model complexity. Although several authors have stressed the importance of inspecting the hyperparameters because default settings did not always yield an optimal performance (Elith et al., 2010; Merow, Smith, & Silander, 2013; Warren & Seifert, 2011; Warren, Wright, Seifert, & Shaffer, 2014), the time‐consuming task of comparing models trained with a multitude of possible combinations of hyperparameters' values (e.g., Zeng, Low, & Yeo, 2016) may discourage many researchers from doing so in practice.

In order to optimize model complexity and performance, both the predictors used to build the model and the values of hyperparameters should be carefully selected which represents a challenge given the often numerous possible options. The new package *SDMtune* described here offers a framework to build and systematically tune SDMs. The package includes utilities that help R users (R Core Team, 2019) all along the analysis process, from data preparation to graphical representation of the results and reporting. In particular, it contains dedicated functions to perform variable selection and hyperparameter tuning. Hyperparameter tuning, also called hyperparameter optimization, is a process usually based on a trial and error experiment during which several models with different values of the hyperparameters are trained and evaluated in order to identify which combination yields the best performance. The simplest algorithm for hyperparameter tuning, grid search, trains and compares models with all possible combinations of the defined hyperparameters' values and can thus be a very time‐consuming process. While other available R packages contain functions for tuning one (e.g., *ENMeval* (Muscarella et al., 2014), *wallace* (Kass et al., 2018)), *kuenm* (Cobos, Townsend Peterson, Barve, & Osorio‐Olvera, 2019) or several statistical model types (e.g., *biomod2* (Thuiller, Georges, & Breiner, 2019), *sdm* (Naimi & Araújo, 2016), *zoon* (Golding et al., 2018) and *caret* (Kuhn et al., 2019)), functions for data‐driven variable selection are not always included and the hyperparameter tuning is always based on grid search or random search algorithms. *SDMtune* offers an alternative that relies on a genetic algorithm for exploring the hyperparameter configuration space (Lessmann, Stahlbock, & Crone, 2005; Young, Rose, Karnowski, Lim, & Patton, 2015), applicable to the most commonly used SDM algorithms. This method significantly reduces the time required to find a near‐optimal or the optimal model configuration. As an additional advantage, all functions for selecting the variables and tuning the hyperparameters are supported by an interactive real‐time displayed chart that shows the change in model performance during the different steps of function execution. The chart is created in the RStudio (RStudio Team, 2018) viewer pane using the open source library *Chart.js* (https://www.chartjs.org), thus facilitating the understanding of the underlying algorithm action through a graphical representation of the output and avoiding the user's feeling of handling a black box that usually comes up when dealing with complex methods.

## 2 PACKAGE WORKFLOW AND DESCRIPTION

**Figure 1:**
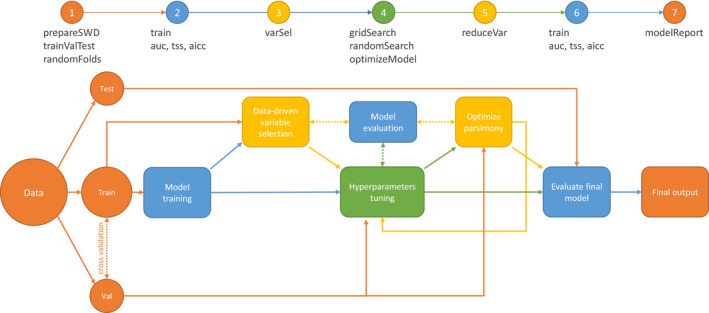
Package workflow illustrating the seven steps of the model tuning process. The functions required to perform the different steps are given in the headline. The different colors indicate different types of steps with: orange: preparation of data and results; blue: model training and evaluation; yellow: variable selection; green: hyperparameter tuning. Dashed connections represent an iterative process

In this section, we present a possible use of the *SDMtune* package that covers a complete analysis in seven steps (Figure 1): (1) preparing data for the analysis; (2) training and evaluating a model; (3) performing variable selection; (4) tuning model hyperparameters; (5) optimizing model parsimony; (6) evaluating the final model; and (7) generating an output report. Users can combine the available functions in a way that best suits them. For example, step 4 could be repeated after step 5 to further fine‐tune model hyperparameters.

### 2.1 Preparing data for the analysis

*SDMtune* uses a special object to compile the data for the analysis. This object, called SWD (samples with data, a format similar to the one used by the Maxent software), bundles all the information related to each record (name of the species, coordinates of the species' presence and absence/background locations, and the values of the environmental variables at these locations), thereby reducing the risk of mistakes in further analyses.

Before starting the analysis the user should decide which evaluation strategy to use. *SDMtune* provides two methods: (1) simple hold‐out validation and (2) k‐fold cross‐validation. The k folds for the cross‐validation can be created either randomly, using the provided **randomFolds** function, or spatially/environmentally clustered, using functions included in the packages *ENMeval* and *blockCV* (Valavi, Elith, Lahoz‐Monfort, & Guillera‐Arroita, 2019): In this case, *SDMtune* will internally convert the folds into the required format. The selected validation strategy is used to perform the variable selection and/or tune the model hyperparameters in order to optimize the model performance and address overfitting. When tuning the hyperparameters, several models with different configurations are trained and evaluated in an iterative process that aims at improving the predictive performance on the validation dataset, or—if cross‐validation is used—on the arithmetic mean of the evaluation metric across all folds. During this process, part of the information contained in the validation dataset is inevitably transferred into the trained model, even if the validation data are not directly used to train the model (Chollet & Allaire, 2018; Müller & Guido, 2016). It is therefore advisable to hold apart an independent partition of the data, that is, the testing dataset, to obtain an unbiased evaluation of the final model (Hastie, Tibshirani, & Friedman, 2009; Merow et al., 2014).

The selection of a metric and a validation strategy should therefore be done early in the model tuning process, because it has implications on how the data should be split before training the first model. Note that the AICc score is computed using all the observation locations (Warren & Seifert, 2011) and does not require to partition the observation data into training and validation.

### 2.2 Training and evaluating a model

Currently, four machine learning methods are available (Table 1): artificial neural networks (ANN), boosted regression trees (BRT), maximum entropy (ME), and random forest (RF). Two different implementations of the ME method can be selected: “Maxent” to use the Java implementation (version 3.4.1 or higher) and “Maxnet” for the R implementation using the *maxnet* package (Phillips, Anderson, Dudík, Schapire, & Blair, 2017; Phillips et al., 2006). There are specific arguments of the **train** function that can be used to set the model hyperparameters. By default, these arguments are set to the same values as implemented in the dependent packages.

**Table 1:** Overview of the hyperparameters that can be tuned per statistical method and underlying package

Method	R package	Hyperparameters	Default value
ANN	*nnet*	Size of hidden layer	–
	(Venables & Ripley, 2002)	Weight decay	0
		Initial random weights	0.7
		Number of iterations	100
BRT	*gbm*	Number of trees	100
	(Greenwell, Boehmke, & Cunningham, 2019)	Interaction depth	1
		Shrinkage	0.1
		Bag fraction	0.5
ME	*dismo*	Feature class combinations	lqph^a^
	(Hijmans, Phillips, Leathwick, & Elith, 2017)	Regularization multiplier	1
		Number of iterations	500
	*maxnet*	Feature class combinations	lqph
	(Phillips, 2017b)	Regularization multiplier	1
RF	*randomForest*	Number of randomly sampled variables	floor(sqrt(#variables))
	(Liaw & Wiener, 2002)	Number of trees	500
		Minimum size of terminal nodes	1

Note

The meaning of each hyperparameter can be found in the respective package documentation and default values, when available, are provided in the last column.

^a^ (l) linear, (q) quadratic, (p) product, and (h) hinge.

A trained model can be evaluated using one of the three implemented metrics: (1) the area under the receiver operating characteristic (ROC) curve (AUC) (Fielding & Bell, 1997), (2) the true skill statistic (TSS) (Allouche, Tsoar, & Kadmon, 2006), and (3) Akaike's information criterion corrected for small sample sizes (AICc, only for ME method) (Burnham & Anderson, 2004; Warren & Seifert, 2011). It should be noted that AICc is a relative measure describing the model fit in relation to complexity (parsimony) but holds no information on predictive performance. It can thus only be used to compare competing models trained using the same data but not for final model evaluation.

### 2.3 Performing the variable selection

When the environmental variables used to train the model are highly correlated, it is difficult to interpret the model output, especially the relative importance of the variables and their response curves. A common practice is thus to select a subset of variables among which collinearity falls below a predefined threshold. A reasonable approach to remove highly correlated variables is to base the selection on expert knowledge, that is, retaining the environmental variable that is most likely to be ecologically meaningful for the target species. When this is unknown, an alternative approach is a “data‐driven” variable selection that uses the information contained in the data to select the variable with the highest explanatory value among those that are highly correlated. The function **varSel** iterates through several steps: Starting from a trained model, it checks if the variable ranked as the most important (using the permutation importance or, optionally for Maxent models, the percent contribution (Phillips, 2017a)) is correlated with any of the other variables, using a given correlation criterion (e.g., Spearman's rho) and correlation threshold. If so, a leave‐one‐out Jackknife test is performed, starting with the full model, and among all correlated variables the one that decreases least model performance on the training dataset is discarded. A new model without this variable is then trained and again checked for highly correlated variables. The process is repeated until the correlations among all retained variables fall below the predefined threshold. During the execution of the function **varSel**, a real‐time chart shows which variable is removed and the relative effect on the model performance.

### 2.4 Tuning the model hyperparameters

Tuning the model hyperparameters is a long process, as it requires testing many combinations of the hyperparameters in order to identify the best performing model. The simplest tuning method, known as “grid search,” is implemented in the function **gridSearch**. The user has the possibility to define a set of possible values for one or several hyperparameters, out of which the function will create all possible combinations. The function also returns the value of the chosen evaluation metric so that the user can see the effect of varying the hyperparameters on the model performance.

**Figure 2:**
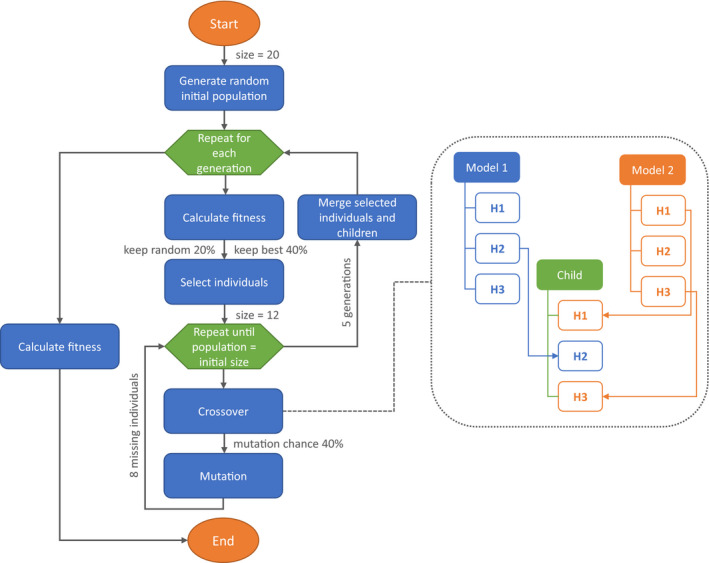
Flowchart illustrating the steps of the genetic algorithm implemented in the function **optimizeModel**, with orange ovals representing the begin and the end of the algorithm, blue boxes the main operations executed by the algorithm, and the green hexagons the iteration loops. In gray are provided the default values used by the function, with “size” indicating the initial population size; “keep best” the proportion of best models retained; “keep random” the proportion of less performing models retained; “mutation chance” the probability that a mutation event occurs. Keep best and keep random are provided as proportion of the initial population size. The dotted box shows an example of crossover during which two models, randomly selected from the selected "individuals", are combined to generate a child model that inherits the first and third hyperparameters' values from Model 2 and the second from Model 1. When the number of generations is zero, the flowchart represents the algorithm implemented in the function **randomSearch**

Grid search is based on a brute force method that results in a very time‐consuming process with high computational costs. A possible alternative is to randomly select some hyperparameters' combinations among the user‐defined values (Bergstra & Bengio, 2012). This approach is implemented in the **randomSearch** function that usually finds a better performing model compared with the starting one. However, the disadvantage of the grid search and random search methods is that they do not use any information acquired during the iteration through the hyperparameter configuration space in order to improve the model performance. The function **optimizeModel** applies a genetic algorithm (Holland, 1992) instead, to more quickly optimize the combination of the hyperparameters (an example of a genetic algorithm used to define hyperparameters and architecture of a deep neural network is presented by Miikkulainen et al. (2018)). The algorithm (Figure 2) starts by generating a random initial “population” of models (using the **randomSearch** algorithm), with a given “population size". The “fitness” of the population is measured with the chosen evaluation metric computed on the validation dataset and models are ranked accordingly. During the evaluation of the “fitness,” underfitting is controlled by ensuring that models for which the evaluation metric computed for the validation dataset is higher than the one computed for the training dataset are ranked in the last positions. At this point starts, the selection process during which some models (“individuals”) are selected according to their “fitness” from the initial “population” to create the first “generation.” There are two selection criteria. At first, a predefined proportion of the “fittest” models (i.e., models ranked in the first positions) is retained. Afterward, a small portion of the poor performing models (i.e., those not selected as “fittest”) is randomly retained in order to keep more variation in the population and reduce the possibility that the algorithm falls in a local optimum. The retained models are then submitted to the optimization process: they are “bred” (i.e., combined) to create other "individuals" and to reach again the predefined “population” size. In this process, two models, called “parents,” are randomly selected from the retained models (“selected individuals”) to “breed” and generate a “child.” This new model will randomly inherit a value for each hyperparameter from one of the “parents,” a process called “crossover.” A “mutation” chance with a predefined probability is added to increase the variation in the population. When the “mutation” is triggered one of the model's hyperparameter is randomly selected and its value is randomly sampled from those available but not included in the “parents.” Once the population reaches the defined size, the “fitness” is calculated again, and the process is repeated for the number of generations specified in the function. The user can set all the arguments: population size, number of generations, fractions of both best and worst performing models to be retained at each generation as well as the probability of mutation during crossover episodes, but default values—that will work in most cases—are also defined. All the functions described in this section come with a real‐time chart showing the model performance while the algorithm is running in the background.

### 2.5 Optimizing model parsimony

As soon as an optimal hyperparameter combination has been selected, we may want to reduce model complexity by removing some environmental variables ranked as less important. The function **reduceVar** automates this with two alternative approaches: (a) removing all the variables with an importance lower than a given threshold in a stepwise fashion, starting from the variable with the lowest importance; (b) removing the variables only if the model performance does not decrease compared to the initial model, according to a given evaluation metric. In the second case, a leave‐one‐out Jackknife test is performed. If removing one of the variables ranked below the given threshold does not decrease the performance of the model on the validation dataset compared to the initial model, that variable is discarded. A new model, trained without this variable, is checked again with the Jackknife test, and the process is repeated until all the variables with an importance lower than the given threshold are either retained or discarded. A real‐time chart showing the removed variable together with its relative effect on model performance is generated during the execution of the function.

### 2.6 Evaluating the final model

At this point, after the variable set has been optimized (**varSel** and **reduceVar**) and the hyperparameters of the model have been tuned (**gridSearch**, **randomSearch**, or **optimizeModel**) the model can be evaluated on the held apart testing dataset, which was never used during the tuning procedure, using one of the functions that compute the chosen metric (i.e., AUC or TSS). Another possibility would be to train a new model using the selected variables and hyperparameter combinations with the full dataset (i.e., without applying cross‐validation or data partitioning) and evaluate it on the held apart testing dataset (Chollet & Allaire, 2018). This way the model can avail of a greater amount of information and might thus be able to generalize better.

### 2.7 Creating the output

There are several functions for visualizing the model results and predictions. The user can plot the response curves, the variable importance, the ROC curve, project the predicted relative probability of species occurrence to the extent of the environmental variables, plot and save the results as a map with either continuous (relative occurrence probabilities) or binary (presence‐absence) values based on various threshold values. *SDMtune* implements its own algorithm to make predictions for “Maxent” models without calling the Java software. This results in a much faster execution that allows considerably speeding up projections, which is particularly useful when models are evaluated using the AICc, as this metric requires computing the Maxent raw output for the full geographic extent of the environmental variables. The prediction produced by our algorithm may—in some cases—differ marginally from the output of the Java implementation, which could result in only slightly different prediction values. Finally, the **modelReport** function creates a report similar to the one produced by the Maxent software, for all methods.

## 3 PERFORMANCE ASSESSMENT OF GENETIC ALGORITHM

We evaluated the performance of the genetic algorithm in terms of time‐saving and model accuracy for the four SDM‐methods available in *SDMtune* by comparing the output of the **optimizeModel** and **gridSearch** functions. We used the **virtualSp** dataset provided with the package. This dataset contains simulated presence, absence, and background locations generated with the package *virtualspecies* (Leroy, Meynard, Bellard, & Courchamp, 2016). For artificial neural network, boosted regression trees, and random forest we used the presence and absence datasets, while for the maximum entropy method we used the presence and background datasets. The maximum entropy method was performed with the “Maxnet” implementation. In all cases, a 10‐fold cross‐validation was used as validation strategy and the AUC was used as evaluation metric. As first step, we trained a model with default hyperparameters' values (for artificial neural network we used an inner layer of a size equal to the number of environmental variables), and then executed the two functions testing 1200 possible hyperparameters' combinations (Table A1, for the **optimizeModel** function we used default arguments). The results of the analysis are presented in Table 2. In all cases, the **optimizeModel** functions found a near‐optimal solution in a significantly reduced amount of time.

**Table 2:** Performance assessment of the **gridSearch** compared to the **optimizeModel** function for model tuning regarding execution time (expressed as HH:MM:SS) and evaluation metric (on the training dataset “Train AUC,” the validation dataset “Val AUC,” given as arithmetic mean across the folds of a 10‐fold cross‐validation) for the four methods implemented in *SDMtune*

Method	Default model		Genetic algorithm			Grid search		
	Train AUC	Val AUC	Train AUC	Val AUC	Time	Train AUC	Val AUC	Time
ANN	0.8600	0.8619	0.9839	0.9590	00:11:44	0.9814	0.9615	05:51:33
BRT	0.9873	0.9750	0.9905	0.9779	00:01:33	0.9892	0.9787	00:29:45
RF	1	0.9724	1	0.9740	00:02:16	1	0.9735	00:48:03
Maxnet	0.8681	0.8561	0.8710	0.8565	00:17:49	0.8702	0.8567	05:01:21

Note

Models were trained using the **virtualSp** dataset available with the package and 1200 possible hyperparameters' combinations. Presence and background locations were used for the Maxnet method, presence and absence locations for the other methods.

## 4 EXAMPLE OF APPLICATION: BEARDED VULTURE IN THE SWISS ALPS

To demonstrate possible applications of *SDMtune,* we used 1947 observation locations of the Bearded vulture (*Gypaetus barbatus*) collected in Switzerland between 2004 and 2017. The occurrences were gathered from two databases: the International Bearded Vulture Monitoring (IBM) database and ornitho.ch, the official birding exchange platform in Switzerland. Clumped observations were removed with a spatial thinning of 250 m using the *spThin* package (Aiello‐Lammens, Boria, Radosavljevic, Vilela, & Anderson, 2015). We randomly split the observations into two partitions and used 80% (1363 observations) as training dataset and the remaining 20% (584) as testing dataset. A set of 39 environmental predictors that might be relevant for the species was prepared for the analysis, as using numerous predictors together with a large amount of species observations allows for a better illustration of the advantages and time‐saving functionalities provided by our package. The variables included information on topography, climate, geology, anthropogenic infrastructure, land cover, and food availability, referring to Hirzel et al. (2004). All predictors were prepared as raster maps with a resolution of 100 × 100 m, with each cell containing the average value of the respective variable within a 1 km^2^ circular moving window (a list of the variables is provided in Appendix A, Table A2). The whole analysis was conducted using R version 3.6.0 (R Core Team, 2019).

We performed the data‐driven variable selection using the function **varSel** on the initial set of 39 predictors. As a first step, we trained a model using the “Maxent” method with default settings (i.e., linear, quadratic, product and hinge as feature class combinations, regularization multiplier equal to 1, 10,000 background locations and 500 iterations) and the 39 environmental variables. We then used the **varSel** function to execute the variable selection using the percent contribution to rank variable importance and the AUC as performance metric. The function arguments were set to check for Spearman's correlation coefficients |*r*_s_| greater than or equal to 0.7, based on 30,000 random background locations (Table A3).

**Figure A1:**
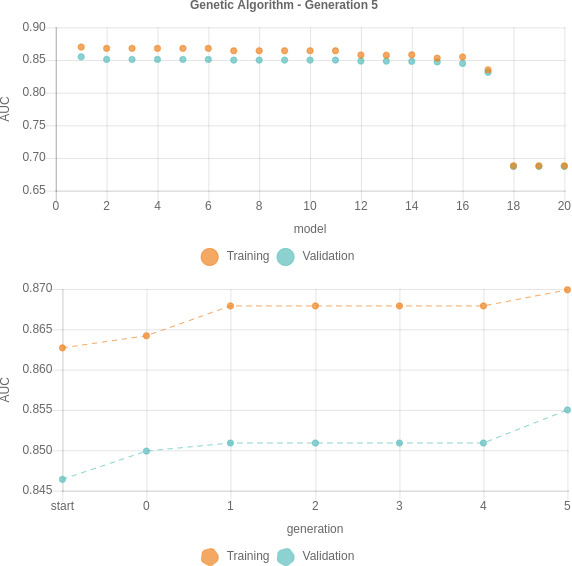
Snapshot of the real‐time chart after executing the **optimizeModel** function (with a “population size” of 20 models and five model generations) using 1200 different hyperparameter combinations on the case example data of the Bearded vulture. The scatterplot on top shows the training AUC (in orange) and the validation AUC (in blue) of the 20 ranked models at the end of the fifth generation, given as the arithmetic mean of the fourfold cross‐validation. The line plot at the bottom shows the increase in model performance (based on the AUC on both training and validation dataset) at each generation with start: the starting model before running the optimization process, 0: the best performing model after the random population is created, 1–5: the best performing models in each of the five generations of the optimization process

Starting with the model trained using the 28 selected variables (i.e., the output of the **varSel** function, Table A4), we conducted a simple experiment to investigate the performance of the **optimizeModel** compared to the **gridSearch** function in terms of execution time and best hyperparameter combination. We selected the AUC as the performance metric running a fourfold cross‐validation. The folds were created by randomly splitting the training dataset with the function **randomFolds**. For the **optimizeModel** function, we used the default arguments: a population size of 20 models, five generations, kept 40% of the best performing models, randomly retained 20% of the less performing ones and used a mutation chance of 40%. We tested different sets of hyperparameters (Table A5 and Figure A1), varying the feature class combinations, the regularization multiplier and the number of iterations. The results illustrate how using the **optimizeModel** function tremendously reduces computation time while providing a near‐optimal solution when the number of hyperparameter combinations increases (Table 3). In our experiment, with 1200 possible hyperparameter combinations, the execution time dropped from 21 hr 14 min and 45 s using **gridSearch** to 1 hr 6 min and 58 s using **optimizeModel** with a similar predictive performance of the resulting models (mean validation AUC across the fourfold of 0.8588 and 0.8550, respectively).

**Table 3:** Performance of the **gridSearch** compared to the **optimizeModel** function for model tuning regarding execution time (expressed as HH:MM:SS) and evaluation metric (on the training dataset “Train AUC,” the validation dataset “Val AUC” and the difference between both “Diff AUC,” given as arithmetic mean of the fourfold cross‐validation) on the case example data of the bearded vulture

h	Algorithm	Exec. time	Train AUC	Val AUC	Diff AUC	FC^a^	reg	iter
75	gridSearch	01:29:24	0.8687	0.8581	0.0106	lqph	3.0	500
	optimizeModel	01:06:50	0.8687	0.8581	0.0106	lqph	3.0	500
150	gridSearch	02:29:16	0.8687	0.8581	0.0106	lqph	3.0	500
	optimizeModel	01:16:25	0.8687	0.8581	0.0106	lqph	3.0	500
300	gridSearch	04:56:44	0.8691	0.8584	0.0107	lqph	2.9	500
	optimizeModel	01:15:09	0.8691	0.8581	0.0110	lqph	2.8	500
600	gridSearch	10:57:43	0.8707	0.8588	0.0119	lqph	2.7	700
	optimizeModel	01:18:46	0.8707	0.8588	0.0119	lqph	2.7	700
1200	gridSearch	21:14:45	0.8706	0.8588	0.0118	lqph	2.8	900
	optimizeModel	01:06:58	0.8700	0.8550	0.0149	lqph	1.9	700

Note

The models were trained using the Maxent method.

The number of tested hyperparameters' combinations is given by “h”. A description of the exact hyperparameters' combinations is provided in Appendix A, Table A5. “FC” represents the feature class combination, “reg” the regularization multiplier and “iter” the number of iterations for the best performing model.

^a^ FC: (l) linear, (q) quadratic, (p) product, and (h) and hinge.

**Figure A2:**
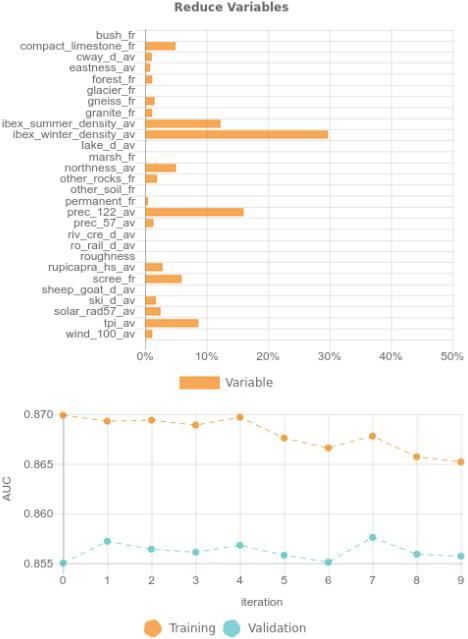
Snapshot of the real‐time chart after executing the **reduceVar** function on the case example data of the Bearded vulture. The bar chart on top shows the 28 uncorrelated environmental variables with the percent contribution of the retained environmental variables at the end of the selection process, calculated according to Phillips et al. (2006). The line chart at the bottom shows the change in model performance (based on the AUC on both the training and validation dataset, given as arithmetic mean of the fourfold cross‐validation) at each iteration where a single variable is removed. In the RStudio viewer pane, the chart is interactive and when the user hovers over the line or bar chart a tooltip reports the variable that has been removed and the relative model performance values. Variable codes are provided in Table A2

In a next step, we investigated whether the final evaluation of the resulting models provided similar results. For this purpose, we selected the models with the optimized hyperparameters' combination (i.e., the output of the **optimizeModel** and **gridSearch** functions run with 1200 different hyperparameters' combinations). We used the **reduceVar** function to test if some variables with low contribution could be removed without affecting the validation AUC. We considered the Maxent percent contribution to rank the environmental variables, a threshold of 2% for variable removal and used the Jackknife approach. We could remove nine and seven environmental variables, respectively, without reducing the mean validation AUC (Table A6 and Figure A2).

Finally, we trained a model using the full training dataset without cross‐validation, the selected environmental variables and the best hyperparameter configuration found by the two functions. We estimated the performance of these tuned models on the held apart testing dataset, obtaining very similar results (Table 4).

**Table 4:** Comparison of model performance between models tuned using the genetic algorithm and grid search implemented in the **optimizeModel** and **gridSearch** function respectively, on the case example data of the Bearded vulture

Step	Dataset	Model performance
Starting model	Training	0.863
	Validation	0.846
	Testing	0.836

		Genetic algorithm	Grid search
Hyperparameter tuning	Training	0.870	0.871
	Validation	0.856	0.859
	Testing	0.848	0.853
Optimize parsimony	Training	0.865	0.868
	Validation	0.856	0.859
	Testing	0.846	0.854
Final model evaluation	Training	0.862	0.867
	Testing	0.846	0.855

Note

Performance is evaluated with the AUC metric on the training, validation and testing datasets as mean AUC of the fourfold cross‐validation at different steps of the modeling workflow. For the final model evaluation the model is trained merging training and validation datasets. The testing dataset refers to the dataset held apart and not used during the hyperparameter tuning and optimize parsimony steps and the starting model is the model trained after removing highly correlated variables.

## 5 DISCUSSION

Most of the algorithms supported by the package have predefined default values for the hyperparameters, while ANN requires the size of the hidden layer to be specified (Table 1). Default values are not necessarily the best choice for any given dataset and modeling problem, and a tuning procedure can improve model performance considerably. For example, the default hyperparameters' values of the Maxent algorithm were derived based on an empirical tuning experiment conducted on 226 species (Phillips & Dudík, 2008), however, several authors found that these values were not always optimal for their specific datasets (Anderson & Gonzalez, 2011; Merow et al., 2013; Radosavljevic & Anderson, 2014; Warren & Seifert, 2011; Warren et al., 2014). While dedicated R packages are available for fine‐tuning Maxent's hyperparameters, like *ENMeval* (Muscarella et al., 2014), *wallace* (Kass et al., 2018), and *kuenm* (Cobos et al., 2019), this process can be very time consuming (Table 2 and 3) and limiting, especially when performed for multiple species. With SDMtune, we introduce a genetic algorithm that drastically reduces the computation time of hyperperameter tuning while achieving an optimal or near‐optimal model configuration.

While the **gridSearch** function can be preferred for tuning a single or a few hyperparameters, it quickly comes to its limits when testing numerous hyperparameters' combinations. In this case, the **randomSearch** function may represent a valid and time‐efficient alternative for finding a better model compared to the one trained with default settings. By taking a random subsample from predefined hyperparameters' combinations, it has to train only *n* models (with *n* equal to the population size, i.e., 20 by default). During this process, it may select the best combination simply by chance. This chance, however, decreases with an increasing amount of possible combinations. The function **optimizeModel**, in contrast, can achieve a better result in a reasonable amount of time, as it makes use of the information generated during the tuning process, thanks to the genetic algorithm. This function also trains a reduced amount of models compared to **gridSearch** with the amount depending on: (1) the population size; (2) the fractions of both best and worst performing models to be retained at each generation; (3) the number of generations, which results in 60 models when using the default settings.

Although there is no rule of thumb to decide when **optimizeModel** should be preferred to **gridSearch** or **randomSearch**, the choice can be supported by considering four important factors. The first and most important factor is the time necessary to train one model, which in turn depends on the sample size, the number of predictors, the selected method, and the setting of the hyperparameters. For instance, increasing the number of trees in RF or BRT increases the computation time as does decreasing the amount of regularization or using hinge or threshold future class combinations in ME methods. The second is the selected evaluation strategy: when k‐fold cross‐validation is performed, the required time to train one model is inflated by the factor k. Moreover, using k‐fold cross‐validation requires to compute the evaluation metric k times and compute their mean value, and this marginally increases the overall computation time. The third factor is the selected evaluation metric. To compute, the AICc is necessary to get the prediction for the whole study area which might take a long time in the case of large extents. The fourth factor is the number of hyperparameters' combinations used for the tuning procedure. Considering these aspects, the total amount of time necessary to tune the hyperparameters with the function **gridSearch** can be roughly estimated based on the time necessary to train and evaluate one model multiplied by the number of possible hyperparameters' combinations. Furthermore, the two functions could also be used in conjunction: the **optimizeModel** function returns *n* models, with *n* equal to the size of the predefined “population” of models (20 by default), ordered by decreasing model performance. The user could inspect the values of the hyperparameters of the returned models and further refine them using **gridSearch**. Finally, in case different hyperparameter‐configurations result in models with equal or similar values of the evaluation metric, the selection of one among the competing models can be based on further exploration, for example, by computing multiple evaluation metrics. It should be stressed that hyperparameter tuning is limited to the values of the hyperparameters defined by the user and thus is not exhaustive. What we defined “best model” refers to the best model among those trained with all the possible combinations of the predefined values. Therefore, the definition of these values determines the quality of the final model.

The genetic algorithm implemented in the function **optimizeModel** relies on some parameters that govern the optimization process (i.e., population size, number of generations, fractions of both best and worst performing models to be retained at each generation, and probability of mutation during crossover episodes), which are provided with default values. We defined these values based on a deep understanding of the algorithm and after testing it multiple times on varying datasets. Nevertheless, although these values could have been hard coded into the source code, we decided to provide a more flexible function making them available as arguments. In the performance assessment of the genetic algorithm and in the example of application presented here (Table 2 and 3), default values worked when testing as much as 1200 predefined hyperparameters' combinations. In case of a similar or higher amount of hyperparameters' combinations, these values might require small adjustments to introduce more variability, for instance by increasing the population size and the probability of mutation.

With the implementation of the genetic algorithm, we introduced a new way of hyperparameters optimization in the field of SDMs. This way could be extended further by testing different modifications. For example, in our implementation only one model is created during the “crossover” event, but two “sibling” models could be produced instead. Furthermore, other optimization algorithms, like the Bayesian optimization, could be implemented. With our **optimizeModel** function, we provide a first implementation of a new algorithm that can be extended in future releases of the package.

Not only the tuning of hyperparameters, but also the selection of environmental variables for SDMs has gained attention in recent years (Jueterbock, Smolina, Coyer, & Hoarau, 2016; Warren et al., 2014; Zeng et al., 2016). Despite the fact that highly correlated environmental variables are not a problem when the aim of the study is prediction in the same extent of the observed data, reducing collinearity is recommended in order to reduce model complexity and increase the interpretability of the predictors (Dormann et al., 2013; Merow et al., 2013). In addition, although the degree of accepted model complexity varies according to the modeling scope(s) (Halvorsen, 2012; Halvorsen, Mazzoni, Bryn, & Bakkestuen, 2015), it has been pointed out that models might perform best when trained with a reduced number of predictors (Brun et al., 2020; Halvorsen et al., 2015). Even though the selection should be driven by the knowledge of the modeled species, this might be difficult when the user must decide among several a priori ecologically relevant predictors for the species, or if the ecology of the species is poorly known. Cobos et al. (2019), with their package *kuenm*, provide a framework that enables tuning several models starting with different sets of environmental variables. Yet, this process still requires predefining the predictor sets. Warren et al. (2014) described a method where environmental variables are removed in a stepwise approach that accounts for regularization tuning, variable importance, and improvements in the AICc metric. A similar approach has been implemented in the package *MaxentVariableSelection* (Jueterbock, 2015), used by Jueterbock et al. (2016) to model the effect of climate change on the Arctic seaweed (*Fucus distichus*). In both examples, all predictors with a contribution‐score lower than a given threshold and predictors highly correlated with the most important variable were removed simultaneously at each step. Given that removing a variable affects the contribution‐score of the remaining predictors and therefore their resulting rank, our functions for data‐driven variable selection remove only one variable at a time. For the same reason, removing highly correlated variables and variables with low contribution is performed by two distinct functions and not combined into the same process, as described in the previous examples. Furthermore, instead of relying merely on a variable's rank of importance for deciding which one to retain, our functions base the selection on a leave‐one‐out Jackknife test, while controlling the desired performance metric. Note that the **varSel** function aims at maintaining the value of the selected metric for the training dataset (i.e., removes the variables that decreases least the evaluation metric) while the **reduceVar** function aims to at least maintain the value of the selected metric for the validation dataset (i.e., removes a variable if the evaluation metric does not drop). The reasons are, first, that highly correlated predictors should be removed before performing any tuning, and second, that optimizing the selected metric for the training dataset allows capturing the information contained in the data, which is especially important if ecological selection criteria are lacking. The over‐ or underfitting can then be controlled later by fine‐tuning the hyperparameters. On the other hand, removing variables with low predictive contribution aims to reduce model complexity and increase model generalization, which is why the validation dataset is used.

There are other R packages which include functions for variable selection. *Caret*, for instance, implements several methods based, among others, on simulated annealing, recursive elimination, or a genetic algorithm. Whereas these methods aim at identifying the best subset of the available variables, our implementations address different problems: **varSel** removes variables to reduce collinearity, and **reduceVar** removes variables that contribute least to the model to increase parsimony. The functions for data‐driven variable selection can be particularly useful when the fitted model is extrapolated in space or time. In such cases, the currently prevailing correlations among the environmental variables may differ from those observed in the new time periods or geographical areas (Braunisch et al., 2013), causing unexpected predictions (Warren et al., 2014). This risk is reduced with a reduced number of predictors. Moreover, reducing the number of predictors may limit overfitting, and thus result in a model that generalizes better and thus yields more accurate predictions for data not used during training. The selection of a threshold to reduce the number of variables with the function **reduceVar** is quite arbitrary. If the aim is to remove as many variables as possible while preserving model performance, the threshold could be set to 100 and the Jackknife method must be selected. On the contrary, if the user, based on his expertise, judges a certain variable as ecologically important for the species and wants to retain it in the model, he could define a threshold that is lower than the importance of this variable. Nevertheless, the functions presented in this article should not be applied blindly. Therefore, *SDMtune* provides interactive real‐time charts to visualize every step of the algorithms with the idea that the user further evaluates the validity of the final output.

These charts are particularly useful for two reasons. First, because they are updated in real time, they confirm that the running function is working properly and is not frozen at some unknown step. This is especially important for functions that take long to be executed. Second, because they are interactive, different types of information can be provided without overloading a single graph, since extra information is embedded in a tooltip that appears when the user hovers over a specific element of the chart. Interactive real‐time charts are well known and used in other fields that represent the state‐of‐the‐art of machine learning, and available in few R packages such as *keras* (Allaire & Chollet, 2020) which allows the user to build complex deep learning models.

## 6 CONCLUSION

The new R package *SDMtune* enables data‐driven variable selection and hyperparameters tuning within a unified and user‐friendly framework. The core functions provide interactive real‐time charts that represent the effect of each step of the implemented algorithms in relation to the model performance and allow a deeper understanding of the automated processes. The new functions we present in this paper (i.e., genetic algorithm for hyperparameter tuning and automated variable selection) are implemented in a framework that also integrates functions already available in other packages. This unification, combining all required functions in a single package, offers the advantage for the user to learn a unique framework instead of jumping from one package to the other, each time having to adapt data structures. Currently, *SDMtune* supports three evaluation metrics (i.e., AUC, TSS, and AICc) and four modeling methods (i.e., ANN, BRT, RF, and ME) and more can be easily added in future releases.

Despite providing comprehensive descriptions and visual illustration of the functions, we still stress that users should be familiar with their data and the selected algorithm used to train their model. Particular attention should be paid to preparing the data before modeling. *SDMtune* also offers functions to prepare the data, but it is upon the user's knowledge and expertise to decide upon the most appropriate way to partition and filter the dataset, accounting for sample size and possible sampling biases, or which metric is best to evaluate the model in relation to the modeling objectives. In this respect Araújo et al. (2019) defined best‐practice standards for SDMs stressing the importance of evaluating models with a temporally or spatially independent dataset (Araújo et al., 2019: Supplement S2.4B). For this reason, *SDMtune* supports functions well developed in other packages (*blockCV* and *ENMeval*) to produce such data partitions. These best‐practices have recently gained importance and have been integrated in the ODMAP standard protocol (Zurell et al., 2020) that provides a workflow for reproducible and good quality analyses.

The package documentation provides a more complete description of all the available functions, and the articles hosted on the package webpage (https://consbiol-unibern.github.io/SDMtune/) describe meaningful examples of application in various fields of ecological research. These examples are also included in the package and accessible through the vignettes.

## 7 INSTALLATION

The package *SDMtune* is available in the CRAN repository at https://CRAN.R-project.org/package=SDMtune and can be installed in R with the command **install.packages(“SDMtune”)**. The package is under development and the source code is hosted in GitHub (https://github.com/ConsBiol-unibern/SDMtune). We encourage future users to provide feedback and report bugs by opening an issue on the GitHub platform.

## CONFLICT OF INTEREST

None declared.

## AUTHOR CONTRIBUTIONS

**Sergio Vignali:** Conceptualization (equal); formal analysis (equal); funding acquisition (supporting); methodology (equal); software (lead); validation (equal); visualization (lead); writing–original draft (lead). **Arnaud G. Barras:** Methodology (supporting); software (supporting); validation (equal); writing–review and editing (equal). **Raphaël Arlettaz:** Funding acquisition (lead); project administration (lead); resources (lead); supervision (supporting); writing–review and editing (equal). **Veronika Braunisch:** Conceptualization (equal); formal analysis (equal); funding acquisition (supporting); methodology (equal); software (supporting); supervision (lead); writing–original draft (lead).

## Data Availability

The Bearded vulture locations and the environmental variables used in the case example have not been archived because part of the data cannot be made publicly available due to data property rights and conservation vulnerability of the species. However, the analysis steps illustrated in the case example could also be reproduced following the articles published on the *SDMtune* website (https://consbiol-unibern.github.io/SDMtune/) and using the **virtualSp** dataset provided with the package. The code necessary to reproduce the performance assessment of the genetic algorithm is provided in Appendix A.

## Appendix A

**Table A1:** Hyperparameter values used during the performance assessment of the genetic algorithm

Method	Hyperparameters' values
ANN	size = 2:81, decay = c(0.01, 0.05, 0.1, 0.3, 0.5), maxit = c(100, 500, 1000)
BRT	n.trees = seq(40, 1020, 20), interaction.depth = 1:4, shrinkage = seq(0.05, 0.1, 0.01)
RF	ntree = seq(420, 1000, 20), mtry = 3:6, nodesize = 1:10
ME (Maxnet)	reg = seq(0.1, 4.88, 0.02), fc = c("l", "lh", "lqp", "lqph", "lqpht")

Note

The values are provided using the R code to generate them.

**Table A2:** Complete list of the environmental variables used in the case example of the Bearded vulture with metric and data source (warm season: May to October; cold season: November to April)

Variable	Abbreviation	Source
Average altitude (m a.s.l.)	altitude_av	DHM^a^
Distance from anthropogenic areas (m)	an_area_d_av	Vector 25^b^
Bush frequency (%)	bush_fr*	Vector 25
Limestone frequency (%)	compact_limestone_fr*	GeoKarten500^c^
Distance from cableways without ski‐lifts (m)	c_way_d_av*	Vector 25
*Sine* of the aspect (−1 to 1)	eastness*	DHM
Forest frequency (%)	forest_fr*	Vector 25
Glacier frequency (%)	glacier_fr*	Vector 25
Gneiss frequency (%)	gneiss_fr*	GeoKarten500
Granite frequency (%)	granite_fr*	GeoKarten500
Ibex density in the warm season (N/ha)	ibex_summer_density_av*	BAFU^d^ + CSCF^e^
Ibex density in the cold season (N/ha)	ibex_winter_density_av*	BAFU + CSCF
Distance from lakes (m)	lake_d_av*	Vector 25
Marshland frequency (%)	marsh_fr*	Vector 25
*Cosine* of the aspect (−1 to 1)	northness_av*	DHM
Open forest frequency (%)	open_forest_fr	Vector 25
Other rock frequency (%)	other_rocks_fr*	GeoKarten500
Grassland and unproductive vegetation frequency (%)	other_soil_fr*	Vector_25 + BfS^f^
Permanent culture frequency (%)	permanent_fr*	Vector 25
Average precipitation in the cold season (mm)	prec_122_av*	WSL^g^
Average precipitation in the warm season (mm)	prec_57_av*	WSL
Distance from rivers and creeks (m)	riv_cre_d_av*	Vector 25
Distance from roads and railways (m)	ro_rail_d_av*	Vector 25
Rock frequency (%)	rock_fr	Vector 25
Distance from rock steeper than 45° (m)	rock45_d_sv	Vector 25 + DHM
Standard deviation (*SD*) of the altitude (m)	roughness*	DHM
Chamois occurrence probability (0–1)	rupicapra_hs_av*	CSCF
Scree frequency (%)	scree_fr*	Vector 25
Sheep and goat warm season density (N/ha)	sheep_goat_d_av*	BsF
Distance from ski‐lifts (m)	ski_d_av*	Vector 25
Slope (degree)	slope_av	DHM
Frequency of slopes steeper than 30° (%)	slope30_fr	DHM
Average solar radiation in the cold season (WH/m^2^)	solar_rad122_av	DHM
Average solar radiation in the warm season (WH/m^2^)	solar_rad57_av*	DHM
Average temperature in the cold season (°C)	tave_122_av	WSL^g^
Average temperature in the warm season (°C)	tave_57_av	WSL
Topographic Position Index^h^ (index)	tpi_av*	DHM
Distance from water (m)	water_d_av	Vector 25
Average wind speed at 100 m above ground (m/s)	wind_100_av*	BFE^i^

Note

Variables were calculated as average value within a circular moving window with *r* = 564 m (1 km^2^) on raster data of 100 × 100 m resolution. The 28 variables selected by the *varSel* function are marked using the * symbol.

^a^ Digital Height Model of Switzerland (Swisstopo):https://shop.swisstopo.admin.ch/en/products/height_models/dhm25.

^b^ Digital Cartographic Model of Switzerland (Swisstopo):https://shop.swisstopo.admin.ch/en/products/maps/national/vector/smv25.

^c^ Geo Maps:https://shop.swisstopo.admin.ch/de/products/maps/geology/GK500.

^d^ Distribution of ibex colonies:https://www.bafu.admin.ch/bafu/de/home/themen/biodiversitaet/zustand/karten.html.

^e^ Centre suisse de cartographie de la faune (CSCF):http://www.cscf.ch/cscf/de/home.html.

^f^ Federal Administration for Statistic Switzerland (BsF):https://www.bfs.admin.ch/bfs/de/home/statistiken.html.

^g^ Federal Institute for Forest, Snow and Landscape Research WSL; available upon request: www.wsl.ch.

^h^ Topographic position index according to Wilson (1984).

^i^ Swiss Wind Atlas (Bundesamt für Energie BFE, 2016).

**Table A3:** Spearman' correlation coefficient of highly correlated environmental variables (*r*_s_ ≤ −0.7 and *r*_s_ ≥ 0.7). For variable codes see Table A2

Var1	Var2	*r*_s_
tave_122_av	tave57_av	0.996
altitude_av	tave57_av	−0.996
altitude_av	tave_122_av	−0.995
forest_fr	open_forest_fr	0.993
slope_av	slope30_fr	0.970
riv_cre_d_av	water_d_av	0.925
rock_fr	rock45_d_av	−0.903
roughness	slope_av	0.882
roughness	slope30_fr	0.863
altitude_av	ro_rail_d_av	0.826
ro_rail_d_av	tave57_av	−0.826
northness_av	solar_rad122_av	−0.814
rock45_d_av	slope_av	−0.814
ro_rail_d_av	tave_122_av	−0.814
rock45_d_av	slope30_fr	−0.813
an_area_d_av	ro_rail_d_av	0.803
solar_rad122_av	solar_rad57_av	0.797
an_area_d_av	tave57_av	−0.774
an_area_d_av	tave_122_av	−0.764
altitude_av	an_area_d_av	0.762
altitude_av	scree_fr	0.738
scree_fr	tave57_av	−0.734
scree_fr	tave_122_av	−0.732
open_forest_fr	tave122_av	0.731
open_forest_fr	tave57_av	0.731
altitude_av	open_forest_fr	−0.718
forest_fr	tave_122_av	0.718
forest_fr	tave57_av	0.718
rock45_d_av	roughness	−0.717
rock_fr	scree_fr	0.707
altitude_av	forest_fr	−0.705

**Table A4:** List of the environmental variables selected by the *varSel* function and relative percent contribution rounded to the first decimal place before executing the *optimizeModel* function

Variable	P. contribution
bush_fr	0.7
compact_limestone_fr	3.5
c_way_d_av	1.1
eastness_av	0.6
forest_fr	0.8
glacier_fr	0.6
gneiss_fr	1.4
granite_fr	1.3
ibex_summer_density_av	9.8
ibex_winter_density_av	41.3
lake_d_av	0.1
marsh_fr	0.0
northness_av	3.9
other_rocks_fr	0.5
other_soil_fr	1.4
permanent_fr	0.8
prec_122_av	11.2
prec_57_av	1.9
riv_cre_d_av	0.4
ro_rail_d_av	0.1
roughness	0.5
rupicapra_hs_av	2.4
scree_fr	4.2
sheep_goat_d_av	1.4
ski_d_av	1.4
solar_rad57_av	0.9
tpi_av	6.7
wind_100_av	1.0

**Table A5:** Hyperparameter values used during the hyperparameter tuning experiment with h: number of tested hyperparameter combinations, FC: feature class combinations with linear (l), quadratic (q), product (p) and hinge (h) feature classes, reg: regularization multiplier and iter: number of iterations

h	FC	reg	iter
75	c(“lq”, “lh”, “lqp”, “lqh”, “lqph”)	seq(0.2, 3, 0.2)	500
150	c(“lq”, “lp”, “lh”, “lqp”, “lqh”, “lqph”)	seq(0.2, 5, 0.2)	500
300	c(“lq”, “lp”, “lh”, “lqp”, “lqh”, “lqph”)	seq(0.1, 5, 0.1)	500
600	c(“lq”, “lp”, “lh”, “lqp”, “lqh”, “lqph”)	seq(0.1, 5, 0.1)	c (500, 700)
1200	c(“lq”, “lp”, “lh”, “lqp”, “lqh”, “lqph”)	seq(0.1, 5, 0.1)	seq (300, 900, 200)

Note

The values are provided using the R code to generate them. In the *optimizeModel* function, in order to have consistent results, we set the *seed* argument to 186,546 (a randomly generated number).

**Table A6:** List of the remaining environmental variables after the execution of the *reduceVar* function (Figure A2) and relative percent contribution rounded to the first decimal place

Variable	Percent contribution	
	*optimizeModel*	*gridSearch*
bush_fr	–	0.9
compact_limestone_fr	7.7	6.8
c_way_d_av	1.7	1.9
eastness_av	1.4	1.4
forest_fr	1.3	1.4
gneiss_fr	1.5	1.7
granite_fr	1.5	1.3
ibex_summer_density_av	19.0	18.5
ibex_winter_density_av	6.8	7.0
northness_av	6.8	6.8
other_rocks_fr	2.2	3.2
other_soi_fr	–	0.8
permanent_fr	0.7	0.7
prec_122_av	24.6	24.2
prec_57_av	2.5	2.4
roughness	1.5	1.0
rupicapra_hs_av	3.5	3.5
scree_fr	9.0	9.9
ski_d_av	1.3	1.3
solar_rad57_av	5.2	3.4
wind_100_av	1.7	1.9

Note

The model parsimony optimization was performed based on the output of the **optimizeModel** and **gridSearch** functions respectively, executed to tune 1200 possible combinations of hyperparameters.

### R code to reproduce the performance assessment of the genetic algorithm

The output presented in the article has been produced using R version 3.6 in a Linux operating system. Results might be slightly different with other versions of R or operating systems due to possible different random numbers generated after setting the seed.

library(SDMtune)

# Set general seed for all experiments

seed = 186546

## Load and prepare data‐‐‐‐‐‐‐‐‐‐‐‐‐‐‐‐‐‐‐‐‐‐‐‐‐‐‐‐‐‐‐‐‐‐‐‐‐‐‐‐‐‐‐‐‐‐‐‐‐‐‐‐‐‐‐‐

files <− list.files(path = file.path(system.file(package = "dismo"), "ex"), pattern = "grd", full.names = TRUE)

predictors <− raster::stack(files)

p_coords <− virtualSp$presence

a_coords <− virtualSp$absence

data <− prepareSWD(species = "Virtual species", p = p_coords, a = a_coords, env = predictors[[1:8]])

folds <− randomFolds(data, k = 10, seed = seed)

## ANN experiment 1200 hyperparameters‐‐‐‐‐‐‐‐‐‐‐‐‐‐‐‐‐‐‐‐‐‐‐‐‐‐‐‐‐‐‐‐‐‐‐‐‐‐‐‐‐‐

# Train starting model ==> size inner layer = number of variables

set.seed(seed)

model_ann <− train("ANN", data = data, size = 8, folds = folds)

auc(model_ann)

auc(model_ann, test = TRUE)

# 1200 hyperparameters' combinations

h_ann <− list(size = 2:81, decay = c(0.01, 0.05, 0.1, 0.3, 0.5),

maxit = c(100, 500, 1000))

nrow(expand.grid(h_ann)) == 1200 # Make sure there are 1200 combinations

# Genetic Algorithm

om_ann <− optimizeModel(model_ann, hypers = h_ann, metric = "auc", seed = seed)

om_ann@results[1:5,]

# Grid Search

set.seed(seed)

gs_ann <− gridSearch(model_ann, hypers = h_ann, metric = "auc", save_models = FALSE)

head(gs_ann@results[order(‐gs_ann@results$test_AUC),])

## BRT experiment 1200 hyperparameters‐‐‐‐‐‐‐‐‐‐‐‐‐‐‐‐‐‐‐‐‐‐‐‐‐‐‐‐‐‐‐‐‐‐‐‐‐‐‐‐‐‐

# Train starting model

set.seed(seed)

model_brt <− train("BRT", data = data, folds = folds)

auc(model_brt)

auc(model_brt, test = TRUE)

# 1200 hyperparameters' combinations

h_brt <− list(n.trees = seq(40, 1020, 20), interaction.depth = 1:4, shrinkage = seq(0.05, 0.1, 0.01))

nrow(expand.grid(h_brt)) == 1200 # Make sure there are 1200 combinations

# Genetic Algorithm

om_brt <− optimizeModel(model_brt, hypers = h_brt, metric = "auc", seed = seed)

om_brt@results[1:5,]

# Grid Search

gs_brt <− gridSearch(model_brt, hypers = h_brt, metric = "auc", save_models = FALSE)

head(gs_brt@results[order(‐gs_brt@results$test_AUC),])

## RF experiment 1200 hyperparameters‐‐‐‐‐‐‐‐‐‐‐‐‐‐‐‐‐‐‐‐‐‐‐‐‐‐‐‐‐‐‐‐‐‐‐‐‐‐‐‐‐‐

# Train starting model

set.seed(seed)

model_rf <− train("RF", data = data, folds = folds)

auc(model_rf)

auc(model_rf, test = TRUE)

# 1200 hyperparameters' combinations

h_rf <− list(ntree = seq(420, 1000, 20), mtry = 3:6, nodesize = 1:10)

nrow(expand.grid(h_rf)) == 1200 # Make sure there are 1200 combinations

# Genetic Algorithm

om_rf <− optimizeModel(model_rf, hypers = h_rf, metric = "auc", seed = seed)

om_rf@results[1:5,]

# Grid Search

gs_rf <− gridSearch(model_rf, hypers = h_rf, metric = "auc", save_models = FALSE)

head(gs_rf@results[order(‐gs_rf@results$test_AUC),])

## Maxnet experiment 1200 hyperparameters‐‐‐‐‐‐‐‐‐‐‐‐‐‐‐‐‐‐‐‐‐‐‐‐‐‐‐‐‐‐‐‐‐‐‐‐‐‐‐

# Train starting model

bg_coords <− virtualSp$background

data <− prepareSWD(species = "Virtual species", p = p_coords, a = bg_coords, env = predictors[[1:8]])

folds <− randomFolds(data, k = 10, only_presence = TRUE, seed = seed)

model_mx <− train("Maxnet", data = data, folds = folds)

auc(model_mx)

auc(model_mx, test = TRUE)

# 1200 hyperparameters' combinations

h_mx <− list(reg = seq(0.1, 4.88, 0.02), fc = c("l", "lh", "lqp", "lqph", "lqpht"))

nrow(expand.grid(h_mx)) == 1200 # Make sure there are 1200 combinations

# Genetic Algorithm

om_mx <− optimizeModel(model_mx, hypers = h_mx, metric = "auc", seed = seed)

om_mx@results[1:5,]

# Grid Search

gs_mx <− gridSearch(model_mx, hypers = h_mx, metric = "auc", save_models = FALSE)

head(gs_mx@results[order(‐gs_mx@results$test_AUC),])
